# Comparison of vacuum-assisted flexible ureteroscopy and percutaneous nephrolithotomy for 2–3 cm upper tract stones: a real-world cohort study

**DOI:** 10.3389/fsurg.2026.1840505

**Published:** 2026-05-20

**Authors:** Xixiang Chen, Xin Huang, Xu Chen, Guo Liu, Liang Wang

**Affiliations:** Department of Urology, Affiliated Xiangxiang People’s Hospital of Changsha Medical University, Xiangxiang, China

**Keywords:** flexible vacuum-assisted ureteral access sheath, percutaneous nephrolithotomy, retrograde intrarenal surgery, stone-free rate, upper urinary tract stones

## Abstract

**Objectives:**

This real-world study aimed to compare the perioperative and recovery outcomes of single-use digital flexible ureterorenoscopy with a flexible-tip vacuum-assisted ureteral access sheath (FV-UAS) against standard percutaneous nephrolithotomy (PCNL) for managing upper urinary tract stones measuring 2–3 cm.

**Patients and methods:**

This single-center, retrospective cohort study enrolled 116 patients (69 FV-UAS, 47 PCNL) between January 2023 and December 2024. The co-primary outcomes were the stone-free rate (SFR) on postoperative day 1 and at 1 month. The SFR on postoperative day 1 was assessed by kidney-ureter-bladder (KUB) radiography or ultrasound, while the SFR at 1 month was assessed by non-contrast computed tomography (NCCT). Secondary outcomes included operative time, length of hospital stay (LOS), hydronephrosis resolution at 90 days, complication rates, and trends in serum creatinine and neutrophil-to-lymphocyte ratio (NLR).

**Results:**

Baseline characteristics were comparable. While the FV-UAS group demonstrated a numerically higher immediate SFR (92.75% vs. 76.59%, *P* = 0.0132), this finding should be interpreted as exploratory due to the limited sensitivity of KUB/ultrasound for early residual fragment detection. The stone-free rate at 1 month, assessed by NCCT, was excellent and comparable between groups (98.55% vs. 91.49%, *P* = 0.1564). Operative time (64.0 ± 9.3 vs. 72.3 ± 8.8 min, *P* = 0.0048) and LOS (3.5 vs. 6.2 days, *P* < 0.0001) were shorter in the FV-UAS group, which also had lower peak postoperative pain scores and body temperature. The rise in serum creatinine was slightly lower in the FV-UAS group, though the clinical significance of this small absolute difference is uncertain. The overall complication rate (Clavien-Dindo Grade I-V) was markedly lower for FV-UAS (10.1% vs. 40.4%, *P* < 0.001). Rates of complete hydronephrosis resolution at 90 days were comparable (94.20% vs. 87.23%, *P* = 0.3863).

**Conclusion:**

In this real-world study, the FV-UAS technique for 2–3 cm upper tract stones was associated with comparable outcomes and stone clearance compared to PCNL. It offered excellent stone clearance, faster recovery, reduced postoperative pain and inflammation, and a more favorable safety profile, particularly regarding bleeding. FV-UAS represents a compelling minimally invasive alternative for selected patients in this stone size category.

## Introduction

Urinary stone disease represents a prevalent and burdensome condition worldwide, with a lifetime risk exceeding 10% and increasing incidence linked to dietary and metabolic factors (1–3). Upper urinary tract stones, encompassing renal and proximal ureteral calculi, pose significant clinical challenges due to their potential to cause obstruction, infection, renal colic, and, if untreated, progressive renal damage (4). The management paradigm for these stones has evolved significantly over recent decades, shifting from open surgery to minimally invasive endourological techniques, which aim to achieve high stone-free rates (SFR) while minimizing patient morbidity and preserving renal function (5). Recent advancements continue to refine these techniques, particularly percutaneous nephrolithotomy (PCNL), which remains a cornerstone of treatment for larger stones despite its inherent invasiveness (6).

The choice of surgical intervention is guided by stone characteristics (size, location, density, composition), renal anatomy, patient comorbidities, and surgeon expertise. For larger upper tract stones (typically >2 cm), PCNL has long been considered the “gold standard” treatment, offering excellent SFRs in a single session (7). The procedure involves creating a percutaneous tract into the renal collecting system, allowing for the use of larger instruments to fragment and remove stones. However, PCNL is not without significant drawbacks. It is an invasive procedure associated with non-negligible risks, including bleeding requiring transfusion (in up to 7%–18% of cases), adjacent organ injury, pleural effusion, and the almost universal need for a nephrostomy tube, which contributes to postoperative pain and prolonged hospitalization (8–10). Furthermore, the creation of a renal parenchymal tract inherently carries a risk of long-term renal scarring and potential functional loss, a concern particularly relevant for patients with pre-existing renal insufficiency or those who may require future repeated interventions (11, 12).

In contrast, retrograde intrarenal surgery (RIRS) using flexible ureterorenoscopes has emerged as a compelling alternative, especially for stones 1–2 cm in diameter, offering a truly minimally invasive transurethral approach that avoids parenchymal puncture (13). Its advantages include lower major complication rates, reduced postoperative pain, shorter hospital stays, and faster convalescence. The technological refinement of digital flexible ureteroscopes, high-power holmium lasers, and improved accessory devices has steadily expanded the indications for RIRS (14, 15). However, when applied to larger stone burdens, conventional RIRS faces its own set of limitations. The standard ureteral access sheath (UAS), while facilitating irrigation and scope passage, has a fixed, rigid design that may not conform optimally to the tortuous ureter, potentially increasing the risk of ureteral wall injury (16). More critically, the confined working channel of the flexible scope limits simultaneous suction, leading to the accumulation of stone dust and fragments during laser lithotripsy. This “snow-globe” effect impairs visualization, prolongs operative time, and may necessitate multiple instrument passages, increasing trauma and procedural complexity. Moreover, the continuous irrigation required to maintain vision can lead to elevated and fluctuating intrarenal pressures (IRP). Sustained high IRP is a well-documented cause of pyelovenous and pyelolymphatic backflow, which is implicated in postoperative fever, systemic inflammatory response, and potentially urosepsis (16, 17). Thus, the quest for innovation in RIRS has focused on improving clearance efficiency and mitigating rises in IRP during prolonged procedures for larger stones.

The recent development of the flexible-tip, vacuum-assisted ureteral access sheath (FV-UAS) represents a technological advancement designed to address these specific shortcomings of conventional RIRS (18). This device integrates a flexible, deflectable distal tip intended to reduce ureteral trauma during insertion and navigation. More importantly, it incorporates a dedicated suction port connected to an intelligent pressure-regulated aspiration system. This system allows for active, continuous, or intermittent evacuation of irrigation fluid, stone debris, and blood clots during lithotripsy, theoretically maintaining a clear visual field, facilitating the “dusting-and-suction” technique, and actively regulating IRP within a safer range (19). However, the evidence remains nascent. There is a conspicuous paucity of robust, comparative real-world studies directly pitting this novel FV-UAS technique against the established benchmark, PCNL, for stones in the 2–3 cm range, a clinical “gray zone” where the optimal surgical strategy is most debated.

Therefore, a rigorous, head-to-head comparison evaluating not only efficacy but also comprehensive safety and functional recovery metrics is urgently needed to define the potential role of FV-UAS in the therapeutic arsenal. Most existing reports on FV-UAS are small, single-arm case series or compare it only to standard RIRS, lacking the contextual benchmark of PCNL (20). Furthermore, assessments often focus on immediate perioperative outcomes, with limited data on inflammatory response, renal functional trajectory, and long-term anatomical recovery, such as hydronephrosis resolution.

This real-world, comparative cohort study was thus conceived to fill this evidence gap. We aimed to systematically evaluate the efficacy and safety of single-use digital flexible ureterorenoscopy combined with a FV-UAS vs. standard PCNL for the management of upper urinary tract stones 2–3 cm in diameter. Our study design captures the nuances of actual clinical practice. We hypothesized that the FV-UAS technique, by enabling more efficient stone clearance and better intrarenal pressure management, would yield non-inferior SFRs while offering advantages in key recovery parameters and potentially a superior safety profile compared to PCNL.

The innovations of this study are twofold: First, it provides a direct comparative analysis of this novel FV-UAS technology against the current gold standard (PCNL) for medium-sized stones in a real-world setting, offering high clinical relevance. Second, beyond conventional efficacy and complication metrics, it incorporates a multidimensional assessment of outcomes, including dynamic trends in systemic inflammatory markers (NLR) and renal function (serum creatinine), as well as long-term anatomical resolution (hydronephrosis), providing a more holistic view of the treatment impact. The findings from this study aim to offer urologists evidence-based insights to refine clinical decision-making for patients with intermediate-sized upper urinary tract stones.

## Methods

### Study design and patient selection

This was a single-center, real-world, retrospective cohort study conducted at Xiangxiang People's Hospital. The study protocol was designed to compare the efficacy and safety of two surgical approaches for the management of upper urinary tract stones: retrograde intrarenal surgery (RIRS) using a single-use flexible ureterorenoscope combined with a FV-UAS vs. standard PCNL. The study was approved by the Institutional Review Board of Xiangxiang People's Hospital, and the requirement for informed consent was waived due to its retrospective nature, in accordance with national regulations and institutional guidelines.

Consecutive patients who underwent surgery for upper urinary tract stones between January 2023 and December 2024 were screened for eligibility. The inclusion criteria were: (1) age between 18 and 75 years; (2) a diagnosis of renal or proximal ureteral stones with a maximum diameter between 2.0 and 3.0 cm, as confirmed by non-contrast computed tomography (NCCT); (3) complete preoperative and postoperative clinical and imaging data. The exclusion criteria were: (1) anatomical anomalies such as horseshoe kidney, pelvic kidney, or duplex collecting systems; (2) active, uncontrolled urinary tract infection or urosepsis at the time of surgery; (3) severe cardiopulmonary comorbidities or bleeding diathesis that contraindicated general anesthesia or surgery; (4) solitary kidney; (5) pregnancy. The final choice of surgical procedure (FV-UAS or PCNL) was made by the attending urologist in consultation with the patient, based on stone characteristics, renal anatomy, and patient preference, reflecting real-world clinical practice. To address potential confounding due to non-randomization, we compared baseline characteristics (Table 1) and found no significant differences. Nevertheless, the possibility of residual confounding cannot be excluded.

**Table 1:** Baseline demographic and clinical characteristics of the study cohort (*n* = 116).

Baseline characteristics	FV-UAS (*n* = 69)	PCNL (*n* = 47)	*P*
Age, (range)	54.0 (50.0–57.0)	53.0 (51.0–56.0)	0.581
Gender, *n* (%)			0.183
Male	45 (65.2%)	24 (51.1%)	
Female	24 (34.8%)	23 (48.9%)	
BMI, kg/m²	25.2 ± 1.0	24.9 ± 0.9	0.174
Stone location, *n* (%)			0.121
Upper calyx	19 (27.5%)	12 (25.5%)	
Lower calyx	13 (18.8%)	17 (36.2%)	
Middle calyx	11 (15.9%)	2 (4.3%)	
Renal pelvis	7 (10.1%)	6 (12.8%)	
Upper ureter	19 (27.5%)	10 (21.3%)	
Maximum stone diameter, mm	26.1 ± 2.6	25.8 ± 2.5	0.536
Stone type, *n* (%)			0.516
Single	31 (44.9%)	24 (51.1%)	
Multiple	38 (55.1%)	23 (48.9%)	
Hydronephrosis severity, *n* (%)			0.383
None	16 (23.2%)	15 (31.9%)	
Mild	15 (21.7%)	5 (10.6%)	
Moderate	22 (31.9%)	14 (29.8%)	
Severe	16 (23.2%)	13 (27.7%)	
Preoperative urinary tract infection, *n* (%)			0.146
No	57 (82.6%)	44 (93.6%)	
Yes	12 (17.4%)	3 (6.4%)	
Serum creatinine, μmol/L	76.2 ± 7.7	75.8 ± 8.1	0.87
Mean Hounsfield Units (HU)	856 ± 112	872 ± 105	0.452
Preoperative NLR	5.0 ± 1.1	5.2 ± 0.8	0.43

FV-UAS, Flexible Vacuum-assisted Ureteral Access Sheath; PCNL, Percutaneous Nephrolithotomy; NLR, Neutrophil-to-Lymphocyte Ratio; BMI, Body Mass Index.

### Surgical techniques

All procedures were performed under general anesthesia by an experienced surgical team led by senior urologists.

#### FV-UAS procedure

Patients were placed in the lithotomy position. Initial diagnostic cystoscopy and retrograde ureteropyelography were performed using a semirigid ureteroscope (8/9.8 Fr) to assess the ureteral orifice and course. A hydrophilic guidewire was then placed in the renal pelvis. Under fluoroscopic guidance, a 12/14 Fr flexible vacuum-assisted ureteral access sheath (PercSys, or equivalent) was advanced over the guidewire. Its flexible tip was positioned at the ureteropelvic junction or within the target renal pelvis. A single-use digital flexible ureterorenoscope was introduced through the sheath. After systematic inspection of the collecting system, stone fragmentation was achieved using a 200 μm holmium:YAG laser fiber (settings: 0.5–1.0 J, 10–20 Hz). The intelligent constant-pressure clearance system connected to the FV-UAS was activated intermittently or continuously during lithotripsy to maintain a clear surgical field, evacuate stone dust and fragments, and regulate intrarenal pressure. The “dusting-and-suction” technique was employed, aiming for complete stone clearance during a single procedure. At the conclusion of the procedure, a 4.8 Fr double-J ureteral stent was routinely placed.

#### PCNL procedure

Patients were initially placed in the lithotomy position for cystoscopic insertion of a 5 Fr open-ended ureteral catheter into the renal pelvis of the affected side. The patient was then turned to the prone position. Renal access was obtained under ultrasound guidance, targeting the intended calyx (preferably a posterior middle or lower pole calyx). After successful puncture, a 0.035-inch J-tip guidewire was coiled in the collecting system. The tract was dilated sequentially using fascial dilators up to 18 Fr or 20 Fr, and a matching Amplatz sheath was placed. A 9.8 Fr rigid nephroscope was introduced. Stone disintegration was performed using a holmium:YAG laser (550 μm fiber) or a pneumatic lithotripter, and fragments were evacuated with forceps or by irrigation. The renal pelvis and all accessible calyces were meticulously inspected for residual fragments. A nephrostomy tube (16 Fr) and a double-J stent were placed at the end of the procedure.

### Data collection and outcome measures

Patient demographics, clinical characteristics, and perioperative data were extracted from the hospital's electronic medical records into a standardized database. Stone density was quantified by measuring the mean Hounsfield Units (HU) of the largest stone on preoperative non-contrast computed tomography (NCCT) images, using a standardized region of interest (ROI) measurement. The co-primary efficacy outcomes were SFR at two timepoints: (1) on postoperative day 1, defined as the absence of residual fragments>2 mm on kidney-ureter-bladder (KUB) radiography or ultrasound; and (2) at postoperative day 30, defined as the absence of residual fragments>2 mm on non-contrast computed tomography (NCCT). Secondary efficacy outcomes included operative time (from start of endoscopy or puncture to stent placement), length of postoperative hospital stay, and the rate of complete hydronephrosis resolution at the 90-day follow-up.

Safety outcomes were comprehensively evaluated and classified according to the Clavien-Dindo classification system. Complications were graded from I to V and categorized as intraoperative or postoperative (within 30 days). Postoperative pain was specifically assessed using the Visual Analog Scale (VAS) and reported as a separate recovery metric, distinct from complications. The 30-day readmission rate was also documented. Specific events like steinstrasse formation and bleeding requiring intervention were documented separately.

To assess the systemic and renal impact, inflammatory and renal functional markers were analyzed. The preoperative NLR was calculated from a complete blood count obtained within 72 h before surgery. Serum creatinine levels were measured preoperatively, within 24 h postoperatively, and at the 3-month follow-up visit.

### Statistical analysis

Statistical analysis was performed using IBM SPSS Statistics for Windows, Version 22.0. Continuous variables were tested for normality using the Shapiro–Wilk test. Normally distributed data are presented as mean ± standard deviation (SD) and were compared between the two independent groups using the independent samples Student's *t*-test. Non-normally distributed data are presented as median (interquartile range, IQR) and were compared using the Mann–Whitney *U*-test. Categorical variables are presented as numbers and percentages (n, %) and were compared using the Chi-square test or Fisher's exact test, as appropriate. All tests were two-tailed, and a *P*-value of less than 0.05 was considered statistically significant.

Furthermore, to adjust for potential confounders inherent to the non-randomized, retrospective design, a multivariable logistic regression analysis was performed to identify independent predictors of the primary outcome (stone-free rate on postoperative day 1). The model included the following covariates: treatment group (FV-UAS vs. PCNL), age, gender, body mass index (BMI), stone location, maximum stone diameter, stone type (single/multiple), hydronephrosis severity, and preoperative urinary tract infection status. The results are presented as adjusted odds ratios (OR) with 95% confidence intervals (CI). Renal pelvis was applied as reference group for stone location.

## Results

### Patient enrollment and baseline characteristics

**Figure 1:**
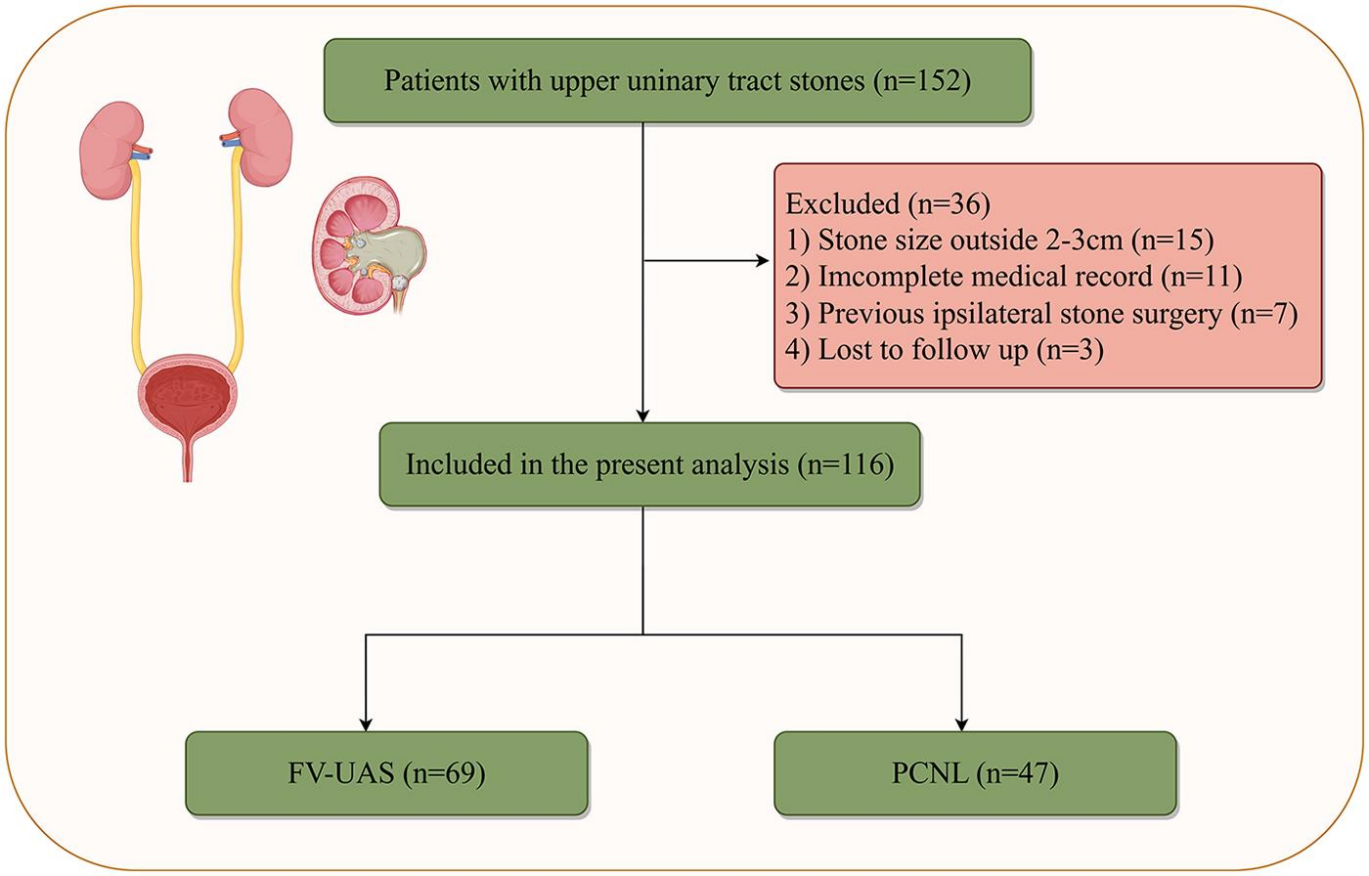
Patient flowchart. Flow diagram detailing the screening, enrollment, and allocation of patients with upper urinary tract stones to the FV-UAS and PCNL treatment groups.

A total of 116 patients diagnosed with upper urinary tract stones were enrolled in this real-world comparative study. The detailed patient selection and allocation process is illustrated in Figure 1. After screening, 69 patients were assigned to undergo stone removal using the FV-UAS technique, while 47 patients underwent the conventional PCNL procedure. The baseline demographic and clinical profiles of the two cohorts are summarized in Table 1. No statistically significant differences were observed between the FV-UAS and PCNL groups regarding age [54.0 [50.0–57.0] vs. 53.0 [51.0–56.0] years, *P* = 0.581], gender distribution (male: 65.2% vs. 51.1%, *P* = 0.183), Body Mass Index (BMI: 25.2 ± 1.0 vs. 24.9 ± 0.9 kg/m², *P* = 0.174), stone location (*P* = 0.121), maximum stone diameter (26.1 ± 2.6 vs. 25.8 ± 2.5 mm, *P* = 0.536), stone type (single: 44.9% vs. 51.1%, *P* = 0.516), severity of hydronephrosis (*P* = 0.383), incidence of preoperative urinary tract infection (17.4% vs. 6.4%, *P* = 0.146; see Table 1 for full data), serum creatinine (76.2 ± 7.7 vs. 75.8 ± 8.1 μmol/L, *P* = 0.87), or preoperative Neutrophil-to-Lymphocyte Ratio (NLR: 5.0 ± 1.1 vs. 5.2 ± 0.8, *P* = 0.43). This baseline homogeneity supports the validity of subsequent outcome comparisons between the two treatment strategies.

### Primary efficacy and long-term functional outcomes

The comparative analysis of the co-primary efficacy outcomes is presented in Table 2. For the early primary endpoint (postoperative day 1 SFR), the FV-UAS group achieved a numerically higher rate, though limited by imaging sensitivity (92.75% vs. 76.59%, *P* = 0.0132; Table 2). This association remained significant after adjusting for potential confounders in a multivariable logistic regression model (Adjusted OR: 3.85, 95% CI: 1.12–13.24, *P* = 0.032; Table 3). For the definitive primary endpoint (postoperative day 30 SFR, assessed by NCCT), the rate was excellent and comparable between groups (98.55% vs. 91.49%, *P* = 0.1564). The distribution of hydronephrosis resolution status (complete resolution, improved, or unchanged) at 90 days postoperatively was comparable between the two groups (94.20% vs. 87.23%, *P* = 0.3863).

**Table 2:** Primary efficacy and long-term functional outcomes (*n* = 116).

Parameters	FV-UAS (*n* = 69)	PCNL (*n* = 47)	*P*
Stone-free rate on postoperative day 1 (%)			0.0132
No residual	64 (92.75%)	36 (76.59%)	
Residual	5 (7.25%)	11 (23.41%)	
Stone-free rate on postoperative day 30 (%)			0.1564
No residual	68 (98.55%)	43 (91.49%)	
Residual	1 (1.45%)	4 (8.51%)	
Complete hydronephrosis resolution at day 90 (%)			0.3863
Complete resolution	65 (94.20%)	41 (87.23%)	
Improved	3 (4.35%)	5 (10.64%)	
Unchanged	1 (1.45%)	1 (2.13%)	

FV-UAS, Flexible Vacuum-assisted Ureteral Access Sheath; PCNL, Percutaneous Nephrolithotomy.

**Table 3:** Multivariable logistic regression analysis for predictors of stone-free rate on postoperative Day 1.

Variable	Adjusted Odds Ratio (95% CI)	*P*-value
Treatment Group (FV-UAS vs. PCNL)	3.85 (1.12–13.24)	0.032
Age (per year)	0.98 (0.92–1.04)	0.521
Gender (Male vs. Female)	1.15 (0.41–3.21)	0.793
BMI (kg/m²)	0.95 (0.62–1.45)	0.812
Stone Location		
Upper Calyx	1.22 (0.31–4.78)	0.774
Middle Calyx	1.05 (0.22–5.01)	0.951
Lower Calyx	0.88 (0.24–3.24)	0.847
Upper Ureter	1.42 (0.38–5.32)	0.602
Maximum Stone Diameter (mm)	0.96 (0.85–1.08)	0.488
Stone Type (Multiple vs. Single)	0.92 (0.33–2.56)	0.873
Hydronephrosis Severity		
Mild	1.10 (0.28–4.32)	0.891
Moderate	0.95 (0.25–3.61)	0.942
Severe	0.82 (0.21–3.20)	0.778
Preoperative UTI (Yes vs. No)	1.55 (0.35–6.89)	0.563

FV-UAS, Flexible Vacuum-assisted Ureteral Access Sheath; PCNL, Percutaneous Nephrolithotomy; BMI, Body Mass Index; UTI, Urinary Tract Infection; CI, Confidence Interval. Reference group for Stone Location: Renal Pelvis.

### Perioperative process and recovery metrics

**Figure 2:**
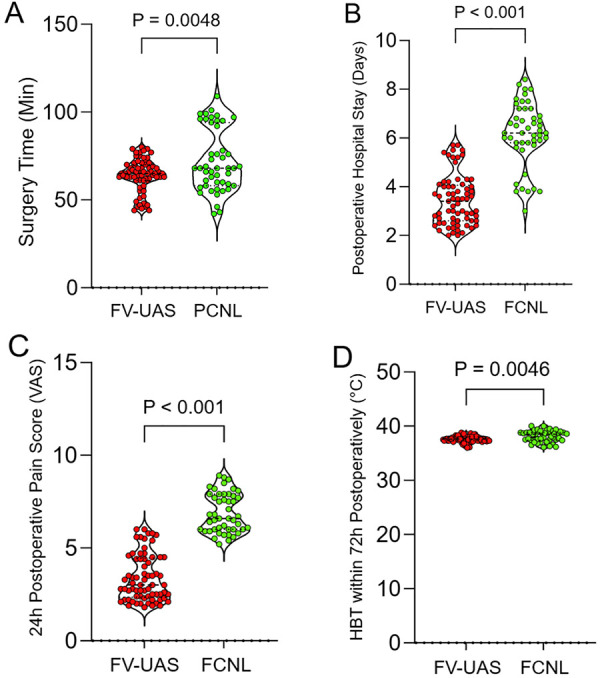
Comparison of perioperative process and recovery metrics. Violin plots comparing **(A)** operative time, **(B)** postoperative hospital stay, **(C)** worst postoperative pain (VAS score) within 24 h, and **(D)** highest body temperature within 72 h between the FV-UAS and PCNL groups. FV-UAS, Flexible Vacuum-assisted Ureteral Access Sheath; PCNL, Percutaneous Nephrolithotomy; VAS, Visual Analog Scale; HBT, Highest Body Temperature.

Key perioperative and recovery parameters are detailed in Figure 2. The FV-UAS procedure was associated with a significantly shorter operative duration compared to PCNL (64.0 ± 9.3 min vs. 72.3 ± 8.8 min, *P* = 0.0048). Consequently, the length of postoperative hospital stay was markedly reduced in the FV-UAS group (3.5 ± 1.0 days vs. 6.2 ± 1.3 days, *P* < 0.0001). Patients in the FV-UAS group also experienced a lower highest body temperature within 72 h postoperatively (37.6 ± 0.6 °C vs. 38.1 ± 1.0°C, *P* = 0.0046) and reported less severe pain, as assessed by the worst Visual Analog Scale (VAS) score within the first 24 h (3.4 ± 1.3 vs. 6.9 ± 1.1, *P* < 0.0001), indicating a milder systemic inflammatory response and superior early postoperative comfort.

### Trends of inflammatory and renal functional markers

**Figure 3:**
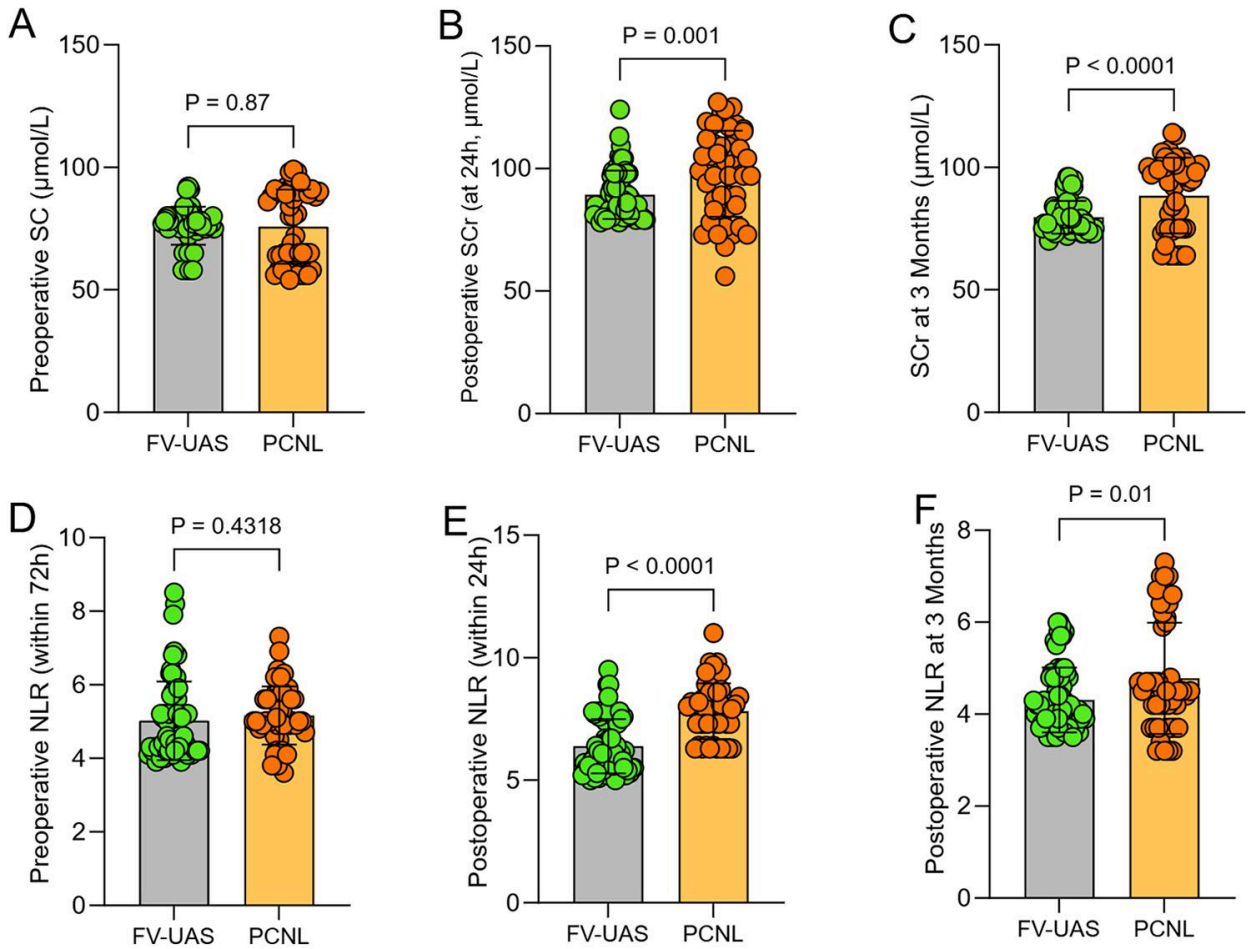
Dynamic trends of inflammatory and renal functional markers. Bar chart illustrating the perioperative trends of **(A–C)** serum creatinine and **(D–F)** Neutrophil-to-Lymphocyte Ratio (NLR) at three time points: within 72 h preoperatively, within 24 h postoperatively, and at 3 months postoperatively. FV-UAS, Flexible Vacuum-assisted Ureteral Access Sheath; PCNL, Percutaneous Nephrolithotomy.

The perioperative and follow-up trends of serum creatinine and NLR are depicted in Figure 3. Preoperatively, serum creatinine (76.2 ± 7.7 vs. 75.8 ± 8.1 μmol/L, *P* = 0.87) and NLR (5.0 ± 1.1 vs. 5.2 ± 0.8, *P* = 0.43) levels were comparable between the FV-UAS and PCNL groups. Within 24 h postoperatively, a more pronounced elevation was observed in the PCNL group for both serum creatinine (89.3 ± 9.8 vs. 97.9 ± 10.5 μmol/L, *P* = 0.001) and NLR (6.4 ± 1.1 vs. 7.8 ± 1.1, *P* < 0.0001). At the 3-month follow-up, while levels in both groups had declined, serum creatinine remained significantly lower in the FV-UAS group (79.7 ± 6.7 vs. 88.5 ± 7.4 μmol/L, *P* < 0.0001). NLR at 3 months was also lower in the FV-UAS group, though the absolute difference was smaller (4.3 ± 0.7 vs. 4.8 ± 0.8, *P* = 0.01).

### Safety outcomes

The comprehensive safety profile, reclassified according to the Clavien-Dindo classification system, is summarized in Table 4. Overall, the incidence of any complication (Clavien-Dindo Grade I-V) was significantly lower in the FV-UAS group (10.1%) compared to the PCNL group (40.4%, *P* < 0.001). This difference was primarily driven by a lower incidence of Clavien Grade I-II complications in the FV-UAS group. The rates of more severe complications (Clavien Grade III-V) were low and comparable between groups. The 30-day readmission rate was 1.4% in the FV-UAS group and 6.4% in the PCNL group (*P* = 0.302).

**Table 4:** Complication outcomes classified by clavien-dindo grade.

Clavien-Dindo Grade	Complication Description	FV-UAS (*n* = 69)	PCNL (*n* = 47)	*P*-value
Any Complication (Grade I-V)		7 (10.1%)	19 (40.4%)	<0.001
Grade I				
	Self-limiting hematuria/Minor bleeding	3 (4.3%)	10 (21.3%)	0.012
	Fever managed conservatively (<38.5°C)	1 (1.4%)	2 (4.3%)	0.578
Grade II				
	Transfusion for bleeding	0 (0%)	1 (2.1%)	0.405
	Antibiotic therapy for urosepsis	2 (2.9%)	4 (8.5%)	0.439
Grade III				
	Ureteral injury requiring stenting	1 (1.4%)	2 (4.3%)	0.578
	Percutaneous drainage for urinoma/abscess	0 (0%)	1 (2.1%)	0.405
Grade IV		0 (0%)	0 (0%)	N/A
Grade V (Death)		0 (0%)	0 (0%)	N/A
Postoperative Pain (VAS >4)	Separate metric, not a complication	2 (2.9%)	5 (10.6%)	0.118
30-day Readmission		1 (1.4%)	3 (6.4%)	0.302

FV-UAS, Flexible Vacuum-assisted Ureteral Access Sheath; PCNL, Percutaneous Nephrolithotomy; VAS, Visual Analog Scale.

## Discussion

This real-world comparative cohort study provides a comprehensive evaluation of a novel surgical approach, single-use digital flexible ureterorenoscopy combined with a FV-UAS, against the established benchmark, PCNL, for managing upper urinary tract stones measuring 2–3 cm. Our findings suggest that the FV-UAS technique is not only effective but may offer several significant perioperative and recovery advantages over PCNL, presenting a compelling minimally invasive alternative in this clinically challenging stone size category.

The management of intermediate-sized (2–3 cm) upper urinary tract stones resides in a therapeutic “gray zone” where the optimal surgical strategy remains debated (5, 13). While PCNL offers high SFR, its inherent invasiveness and associated morbidity are well-documented (7–10). Conversely, conventional retrograde intrarenal surgery (RIRS) for larger stones is hampered by limitations in fragment clearance and the risk of elevated intrarenal pressure (IRP) (16, 17). The FV-UAS system was developed to address these specific shortcomings by integrating active suction to clear debris and regulate IRP (18, 19). This study is conducted to directly compare this evolving FV-UAS technology with standard PCNL in a real-world setting, employing a multidimensional assessment that extends beyond immediate SFR to include inflammatory response, renal functional trajectory, and anatomical recovery. Our results indicate that FV-UAS achieved superior immediate SFR, shorter operative time and hospitalization, reduced postoperative pain and inflammatory response, and a more favorable safety profile, particularly regarding intraoperative bleeding. These findings have significant implications, suggesting that FV-UAS could potentially shift the treatment paradigm for selected patients with 2–3 cm stones towards a less morbid, entirely endoscopic approach without compromising efficacy.

The numerically higher immediate SFR observed in the FV-UAS group (92.75% vs. 76.59%, *P* = 0.0132) must be interpreted with extreme caution. As discussed, the use of KUB/ultrasound on postoperative day 1 is limited by lower sensitivity compared to NCCT, likely leading to an overestimation of true early clearance rates, particularly in the PCNL group, where residual fragments may be more readily missed by less sensitive imaging. Notably, this early difference did not persist at the 30-day follow-up assessed by NCCT (98.55% vs. 91.49%, *P* = 0.1564). This suggests that the apparent advantage of FV-UAS on day 1 is likely an artifact of the imaging modality rather than a true reflection of superior immediate stone clearance. Therefore, the 30-day SFR, assessed by the more sensitive NCCT, represents the definitive measure of efficacy, confirming comparable stone clearance between the two techniques. Crucially, this early advantage did not translate into a long-term difference, as evidenced by the comparable 30-day SFRs assessed by NCCT (98.55% vs. 91.49%, *P* = 0.1564). Therefore, our primary finding should therefore be viewed as an exploratory observation, and the comparable 30-day SFR represents the more definitive measure of efficacy (7). This advantage persisted, though not statistically significant, at the 1-month follow-up (98.55% vs. 91.49%). We attribute this high clearance efficiency of FV-UAS to its integrated vacuum system, which enables continuous or intermittent evacuation of stone dust and small fragments during laser lithotripsy. This “dusting-and-suction” approach mitigates the “snow-globe” effect that impairs visualization and prolongs surgery in standard RIRS, theoretically allowing for more complete intraoperative clearance (18, 19). Recent meta-analyses and comparative studies focusing on RIRS with vacuum-assisted sheaths vs. conventional sheaths or mini-PCNL have reported favorable SFRs, but direct comparisons with standard PCNL are scarce (13, 17, 19). For instance, Deng et al. reported comparable SFRs between vacuum-assisted RIRS and tubeless mini-PCNL for 2–3 cm stones (19). Our study extends this evidence by demonstrating that a modern FV-UAS system can achieve, and in the immediate setting surpass, the clearance efficacy of standard PCNL. In addition, our finding of a significantly higher SFR on postoperative day 1 in the FV-UAS group appears to contradict the long-standing view of PCNL as the gold standard for achieving the highest stone clearance for 2–3 cm stones. We believe this discrepancy can be explained by a combination of factors. First, and most importantly, the limitation of our early imaging modality (KUB/ultrasound) likely led to an overestimation of the true early clearance, as discussed previously. Second, it is plausible that the active suction capability of the FV-UAS system provides a genuine intraoperative advantage in clearing stone dust and small fragments, a phenomenon that may not translate to a long-term difference, as evidenced by the comparable 30-day SFRs. While our multivariable analysis suggests this association is independent of measured confounders, we interpret this exploratory finding with caution. It highlights the potential of this novel technology but does not displace PCNL as a highly effective treatment for this stone size.

Furthermore, the rate of complete hydronephrosis resolution at 90 days was excellent and comparable between groups (94.20% vs. 87.23%, *P* = 0.3863). This indicates that both techniques are effective in relieving obstruction in the long term. The high resolution rate in the FV-UAS group underscores that a minimally invasive, non-puncture approach can achieve anatomical recovery equivalent to that of a percutaneous procedure, allaying concerns that residual fine debris from dusting might perpetuate obstruction. The FV-UAS procedure was associated with a statistically significant reduction in operative time, postoperative hospital stay, peak postoperative fever, and patient-reported pain scores. The shorter operative time (64.0 vs. 72.3 min) is likely multifactorial. The FV-UAS technique eliminates the time-consuming steps of PCNL, such as patient repositioning, renal puncture, tract dilation, and nephrostomy tube placement. Moreover, maintaining a clear visual field via active suction may reduce time spent irrigating and searching for fragments. These findings align with the fundamental premise of minimally invasive surgery, reducing tissue trauma translates to faster recovery (13).

The marked reduction in hospital stay (3.5 vs. 6.2 days) and lower pain scores are direct corollaries of avoiding a renal parenchymal tract and nephrostomy tube, which are significant sources of postoperative pain and morbidity in PCNL (8, 10). The lower highest body temperature in the FV-UAS group suggests a attenuated systemic inflammatory response, which is further supported by the NLR trends.

The postoperative trajectories of serum creatinine and NLR provide objective biochemical insights into the differential impact of the two procedures. We observed a smaller absolute rise in serum creatinine within 24 h postoperatively in the FV-UAS group (89.3 ± 9.8 vs. 97.9 ± 10.5 μmol/L, *P* = 0.001). However, we acknowledge a critical limitation in our renal functional assessment: we evaluated renal function using absolute creatinine values without applying standardized criteria for acute kidney injury (e.g., KDIGO criteria) or calculating the percentage change from baseline. The magnitude of this difference (≤8.6 μmol/L) is small and lacks clear clinical significance in patients with normal baseline renal function. While it may be related to the less invasive nature of the FV-UAS technique, avoiding renal parenchymal puncture and tract dilation inherent to PCNL, this finding should be interpreted with extreme caution. Without standardized classification, the clinical relevance of such minor fluctuations in creatinine remains uncertain, and claims of “renoprotection” are not supported by this study. PCNL involves parenchymal puncture and dilation, which causes localized trauma, bleeding, and inflammation (11). In contrast, FV-UAS is a purely endoluminal procedure, avoiding renal parenchymal violation. The active pressure regulation of the FV-UAS system may also limit pyelovenous backflow of irrigation fluid and potential bacteria/endotoxins, a known contributor to postoperative fever and sepsis (16, 17). Perhaps more importantly, at the 3-month follow-up, serum creatinine levels remained significantly lower in the FV-UAS group. While both groups showed recovery from the acute insult, the FV-UAS group returned closer to its preoperative baseline. This suggests that the avoidance of a parenchymal tract may confer a long-term renal functional advantage, or at minimum, less functional perturbation. This finding aligns with growing concerns about the potential for cumulative renal damage from repeated percutaneous procedures, particularly in patients with recurrent stone disease or pre-existing renal insufficiency (11, 12). Although our study follow-up is mid-term, this functional data is promising and warrants investigation in longer-term studies.

The safety analysis revealed a pronounced advantage for the FV-UAS technique. The overall complication rate (Clavien-Dindo Grade I-V) was markedly lower (10.1% vs. 40.4%, *P* < 0.001), driven primarily by a significant reduction in Clavien Grade I-II complications. This is the most direct evidence of the less invasive nature of FV-UAS. Significant bleeding requiring intervention is the most common significant complication of PCNL, with transfusion rates reported between 7%–18% (7–9). By avoiding renal puncture and tract dilation, FV-UAS inherently eliminates this risk source. Although not statistically significant, the numerical reductions in ureteral/kidney injury and urosepsis in the FV-UAS group are clinically encouraging. The flexible tip of the FV-UAS sheath may reduce ureteral trauma during insertion compared to rigid sheaths (18). Furthermore, by actively controlling IRP and evacuating infected debris, the system may lower the risk of fluid absorption and bacteremia (17, 19).

Notably, events like Steinstrasse and major bleeding were not observed in the FV-UAS cohort but occurred in the PCNL group. The absence of Steinstrasse in the FV-UAS group can be explained by the suction mechanism actively removing smaller fragments, preventing their accumulation in the ureter. The low and comparable postoperative complication and readmission rates further support the safety and tolerability of the FV-UAS approach.

The main intraoperative complications for PCNL in our series (bleeding, injury) are consistent with large registry data (7, 9). For FV-UAS, the complication profile mirrors that of advanced RIRS, with a focus on potential ureteral injury and infection. Our low rate of ureteral injury (1.4%) is comparable to or lower than rates reported for conventional UAS (16), possibly due to the flexible distal tip of the FV-UAS. The 5.8% rate of urosepsis in the FV-UAS group, while lower than PCNL, underscores that even with pressure regulation, RIRS for larger stones carries an infection risk, emphasizing the continued critical importance of preoperative antibiotic prophylaxis and low-pressure irrigation principles.

Our study has several limitations inherent to its real-world, retrospective design. First, the non-randomized assignment of patients introduces potential selection bias, as the final choice of procedure was based on surgeon and patient preference. However, the baseline characteristics were well-balanced, mitigating this concern to some extent. Second, being a single-center study, the results may be influenced by local expertise and protocols; the surgeons were highly experienced, which may limit the generalizability of the excellent outcomes, particularly for the FV-UAS technique which has a learning curve. Third, the sample size, though adequate for detecting differences in primary outcomes, may be underpowered for rare but serious complications. Fourth, while we were able to match groups for stone density (Hounsfield Units), we did not have access to precise stone volume measurements, which is another important aspect of stone complexity. Future studies should incorporate this metric. Furthermore, although we performed multivariable logistic regression for the primary outcome (postoperative day 1 SFR), we did not conduct such adjustments for all secondary outcomes, which represents a limitation of this retrospective analysis. Finally, a detailed cost-effectiveness analysis was not performed, which is an important consideration for healthcare systems given the single-use nature of the digital scopes and FV-UAS system.

In this real-world comparative study, single-use digital flexible ureterorenoscopy combined with a FV-UAS was associated with comparable outcomes and stone clearance compared to standard PCNL for the management of 2–3 cm upper urinary tract stones. The FV-UAS technique achieved excellent stone-free rates, significantly reduced operative time and hospital stay, minimized postoperative pain and inflammatory response, and was associated with a more favorable safety profile, particularly regarding intraoperative bleeding. The technological advancements of active suction and pressure regulation appear to effectively address key limitations of conventional RIRS for intermediate-sized stones. While PCNL remains a crucial and effective tool, particularly for complex or staghorn calculi, our findings suggest that FV-UAS represents a highly effective and less morbid minimally invasive alternative for selected patients with 2–3 cm stones, particularly those seeking a quicker recovery and with favorable anatomy. PCNL remains a crucial option for complex stone configurations or when retrograde access is not feasible. Prospective, randomized trials with larger cohorts and longer follow-up are warranted to confirm these findings and to better define the ideal patient and stone characteristics for this promising approach.

## Data Availability

The original contributions presented in the study are included in the article/Supplementary Material, further inquiries can be directed to the corresponding author/s.
